# β-defensin-4 as an endogenous biomarker in cows with mastitis

**DOI:** 10.3389/fvets.2023.1154386

**Published:** 2023-03-24

**Authors:** Stephan Neumann, Stephan Siegert, Anneke Fischer

**Affiliations:** Institute of Veterinary Medicine, Georg-August-University of Göttingen, Göttingen, Germany

**Keywords:** defensin, mastitis, dairy cow, antimicrobial peptide, biomarker

## Abstract

**Introduction:**

Defensins are peptides with antimicrobial and immunomodulatory effects. Their concentration could be altered during infections and thus provide information on the prognosis and course of the disease. The aim of the present study was to investigate the defensin concentration in cows with mastitis in order to find correlations between clinical expression and course of the disease and the defensin concentration in milk and blood.

**Methods:**

A total of 85 dairy cows were examined. Of these, 30 animals suffered from acute clinical mastitis, 25 animals were diagnosed with subclinical mastitis and 30 animals were considered a healthy comparison group. Beta-Defensin-4 (DEFB-4) was determined by a species-specific enzyme-linked immunosorbent assay (ELISA) (Bovine Defensin Beta 4 ELISA Kit, MyBioSource).

**Results:**

The highest concentrations of DEFB-4 were detected in the animals with acute clinical mastitis. Values of 0 to 895 pg/mL (median: 115 pg/mL) were measured in milk and 40–1,016 pg/mL (median: 245 pg/mL) in serum. The concentrations of this group differed significantly from those of the animals with subclinical mastitis (*p* < 0.0001 serum; *p* = 0.015 milk). In this group, concentrations of 15–211 pg/mL (median: 46 pg/mL) were recorded in milk and 20-271 pg/mL (median: 85 pg/mL) in serum.

**Discussion:**

Our results also show that in cases of acute mastitis after 12 days of treatment there is still an active inflammatory process in the tissue, because no significant reduction of somatic cells and defensin could be found after re-examination. Since the DEFB-4 concentrations of animals with clinical mastitis that had to be treated with antibiotics differed significantly from those of animals with subclinical mastitis that did not require antibiotic treatment, it can be assumed that bovine DEFB-4 is an important endogenous parameter for the defense against bacterial infections of the udder.

## 1. Introduction

The interaction between pathogens and host in an infection-related inflammation is a complex process. Although the understanding of this process is progressing, only a few markers (leukocytes, acute-phase proteins, globulins) have been established to evaluate its extent and course as well as the prognosis. Increasing knowledge of the mechanisms of infection-related inflammation could both generate new markers and open up further therapeutic options in infectious diseases.

Defensins are antimicrobial peptides (AMPs), which are part of the active immune system (1–3). Based on their molecular structure alpha-, beta- and theta-defensins can be distinguished (1, 4). The local release of defensins is induced by a Toll-like receptor (TLR) mediated reaction in which the host cell receptor interacts with pathogen-associated molecular patterns (PAMPs), such as lipopolysaccharides. The consequence of receptor binding is the increased expression of defensins (5, 6). Accordingly, Goldammer et al. (7) could demonstrate an expression of bovine DEFB-5 induced by TLR-2 and TLR-4.

The effects of defensins are antimicrobial and immunomodulatory. The first effect is based on the positive molecular charge, which enables a charge-dependent interaction with the negatively charged bacterial cell wall, especially lipopolysaccharides (8). Thereby, the defensins can cause depolarization of the cell membrane and thus a destabilization of the membrane potential and lysis of the cell (8, 9). Furthermore, defensins show immunomodulatory functions. Some defensins have a chemotactic effect on monocytes and macrophages (10, 11) and induce the activation and degranulation of mast cells (12, 13). Subsequently, histamine and prostaglandin D2 are released, which promote the recruitment of neutrophil granulocytes (14, 15). The degranulation of the recruited neutrophil granulocytes again releases defensins resulting in a positive feedback loop (11). In addition, they act chemotactically on dendritic cells and T-memory cells and thus represent a link between innate and adaptive immune response (16).

The investigation of defensins seems to be interesting in order to identify new endogenous biomarkers for the detection of infection-related inflammatory processes, the prognosis of diseases and possibly to evaluate therapeutic options. Here, mastitis has been chosen as an infectious disease, as it is one of the most common diseases of cows (17–19). It leads to severe financial losses and causes massive use of antibiotics in the dairy industry worldwide (20). Mastitis is usually caused by bacterial infections (21). The main mastitis pathogens are staphylococci, especially *Staphylococcus aureus* (*S. aureus*), streptococci, such as *Streptococcus uberis* (*S. uberis*), as well as enterococci and *Escherichia coli* (*E. coli*) (22).

In the present study, DEFB-4 in cows with mastitis has been investigated. DEFB-4 was chosen because this molecule has already been detected in cows (23–26). Younis et al. (21) have shown that in cows the expression of DEFB-4 genes is induced by *E. coli* infection of the mammary gland. Therefore, it was aimed to determine DEFB-4 protein levels in milk and blood serum in order to investigate possible correlations with acute vs. subclinical mastitis in cows.

## 2. Material and methods

### 2.1. Animals

In the study a total number of 85 cows of the Holstein-Friesian breed were examined.

The animals were divided into three groups. Group 1 is the control group which consisted of clinically healthy cows whose somatic cell count of the milk was ≤ 100,000 cells/mL. None of the udder quarters showed clinical signs of inflammation and the secretion was macroscopically normal.

Group 2 consisted of animals with acute mastitis. Following criteria were used: the somatic cell count in the milk was >100,000 cells/mL and they showed clinical symptoms of inflammation on at least one udder quarter. Furthermore, the milk was macroscopically altered.

Group 3 included animals with subclinical mastitis. Compared to the control group, the somatic cell count in the milk was >100,000 cells/mL. In these animals none of the udder quarters showed clinical signs of inflammation and the milk was not altered macroscopically.

Milk and blood samples were taken from each animal on the first day of treatment and again after 12 days in group 2 and 3. In an additional time kinetics study, four cows with acute mastitis were monitored for seven weeks. They were re-examined after three, five and seven weeks, and one milk and one blood sample were collected each time. One of these cows showed changes in two udder quarters and therefore two milk samples were collected in this case. All examinations were approved by the Lower Saxony State Office for Consumer Protection and Food Safety (Niedersächsisches Landesamt für Verbraucherschutz und Lebensmittelsicherheit, LAVES) (file number: 33.9-42502-05-16A089), carried out in compliance with the Animal Welfare Act and monitored by the Animal Welfare Officer of Georg-August University in Göttingen.

### 2.2. Samples

#### 2.2.1. Milk samples: Preparation for DEFB-4 measurement, somatic cell count, bacteriology

The milk samples were taken after thorough cleaning of the teats with pH skin neutral moist cleaning cloths. In group 2 a milk sample of the inflamed quarter was taken. In group 3 the quarter with the highest somatic cell count accordingly to the California Mastitis Test was sampled, whereas in group 1 milk samples were collected from all quarters. One part of the milk sample was used for DEFB-4 analysis. For this purpose, milk was centrifuged at 1,000 x g for 20 min. After the fat layer was removed, the supernatant was pipetted off, aliquoted into Eppendorf tubes and frozen at −80°C.

Another part of the milk sample was used to measure the somatic cell count using automated fluorescence optical counting (Fossomatic). For this purpose, about five mL of the milk were filled into special tubes for mastitis diagnostics (IfM, Verden, Germany) and sent to the Institute for Milk Testing (Institut für Milchuntersuchung, IfM, Verden, Germany). A third part of the milk sample was used for microbiological examination at the Institute of Microbiology of the University of Veterinary Medicine Hannover by spreading the milk on different media, such as Columbia agar, Gassner agar, selective agar for staphylococci and streptococci, respectively, and adding about 200 μL to a nutrient broth. After specific incubation the colonization levels were divided into low (1.0 x 10^3^ bacteria/mL milk), medium (1.0 × 10^4^ – 10^5^ bacteria/mL milk) and high (≥1.0 × 10^6^ bacteria/mL milk).

#### 2.2.2. Blood samples: Preparation for DEFB-4 measurement, hematology, clinical chemistry

Blood samples were taken from the median caudal vein using a sterile injection cannula (20G 0.9 × 40 mm) and transferred to serum and EDTA tubes (Sarstedt AG & Co, Nümbrecht, Germany). For DEFB-4 analysis one of the serum tubes was centrifuged at 1,000 × g for 20 min, pipetted, aliquoted and frozen at −80°C until analysis.

Furthermore, blood tests were used to detect leukocytes, their differentiation and to measure clinical-chemical parameters to determine the health status of the animals. Hematological examinations were performed on EDTA blood using the IDEXX ProCyte DxTM analysis machine (IDEXX Laboratories Inc., Westbrook, Maine, USA). For clinical-chemical parameters the serum was centrifuged at 1,000 × g for 6 min and photometrically measured by Konelab 20i (Thermo Fischer Scientific Inc., Dreieich, Germany).

### 2.3. Detection of DEFB-4 by enzyme-linked immunosorbent assay

A species-specific quantitative sandwich ELISA kit with a reported sensitivity of 2.0 pg/mL was used to measure DEFB-4 levels in serum and milk (MyBioSource, San Diego, USA; Cat. No.: MBS9353192). The immunogen of the capture antibody is recombinant full length protein of Bovine DEFB-4. The immunogen of the detection antibody is a recombinant fragment according to aa 23-63 of Bovine DEFB-4. The standard is recombinant full-length Bovine protein by E.coli.

According to the manufacturer, the intra- and inter-assay coefficient of variation is <15%, which we were able to confirm with values even <10%. All samples and standards were added in triplicate to the plate pre-coated with a DEFB-4 specific monoclonal antibody. Subsequently, a second antibody conjugated to horseradish peroxidase was added and the plate was incubated for 1 h at 37°C. The plate was washed 4 times with wash solution and all liquid was removed. Hydrogen peroxide and tetramethylbenzidine were added, resulting in a color change to blue during a second incubation period for 15 min at 37°C. The reaction was stopped by adding sulfuric acid, visible by the color change to yellow. The optical density was determined photometrically at a wavelength of 450 nm using a TECAN microplate reader (TECAN Austria GmBH, Grödig, Austria). The standard series was expressed as a linear function, which was then used to calculate the DEFB-4 concentrations.

### 2.4. *In vitro* study

For an *in vitro* study, fresh, untreated cow's milk was collected from four clinically healthy animals and aliquoted into three parts. One sample was left untreated, one sample was inoculated with E. coli (1.0 x 10^3^ CFU) and one sample with Streptococcus (Sc.) agalactiae (1.0 × 10^3^ CFU). The samples were incubated at 37°C and subsamples were analyzed for DEFB-4 after 24, 36, 48, and 72 h. The DEFB-4 concentration used for the time depending comparison was the mean of all four milk samples. In addition, the somatic cell count of the samples was measured (IfM, Verden, Germany).

### 2.5. Statistics

All data was statistically analyzed and graphically presented with the program Prism 8 (GraphPad Software, San Diego, USA).

The values were examined for normal distribution by Shapiro-Wilk test. The median, lower and upper quartile, as well as maximum and minimum values were visualized using box and scatter plots.

Except for the healthy control group, all values of the individual groups were not normally distributed, so non-parametric test procedures were used. For comparison of two independent groups (e.g., healthy/diseased) the Mann-Whitney-U-Test was used, for two interdependent groups (e.g., initial and re-examination of the same animal) the Wilcoxon‘s Signed Rank Test was used. To test for correlations the Spearman rank correlation was performed. A *P* < 0.05 was considered statistically significant. In addition, a ROC analysis was performed to determine the cut-off for distinguishing between subclinical mastitis and healthy cows.

## 3. Results

### 3.1. Comparison of DEFB-4 concentrations of healthy cows vs. cows with acute clinical mastitis

**Figure 1:**
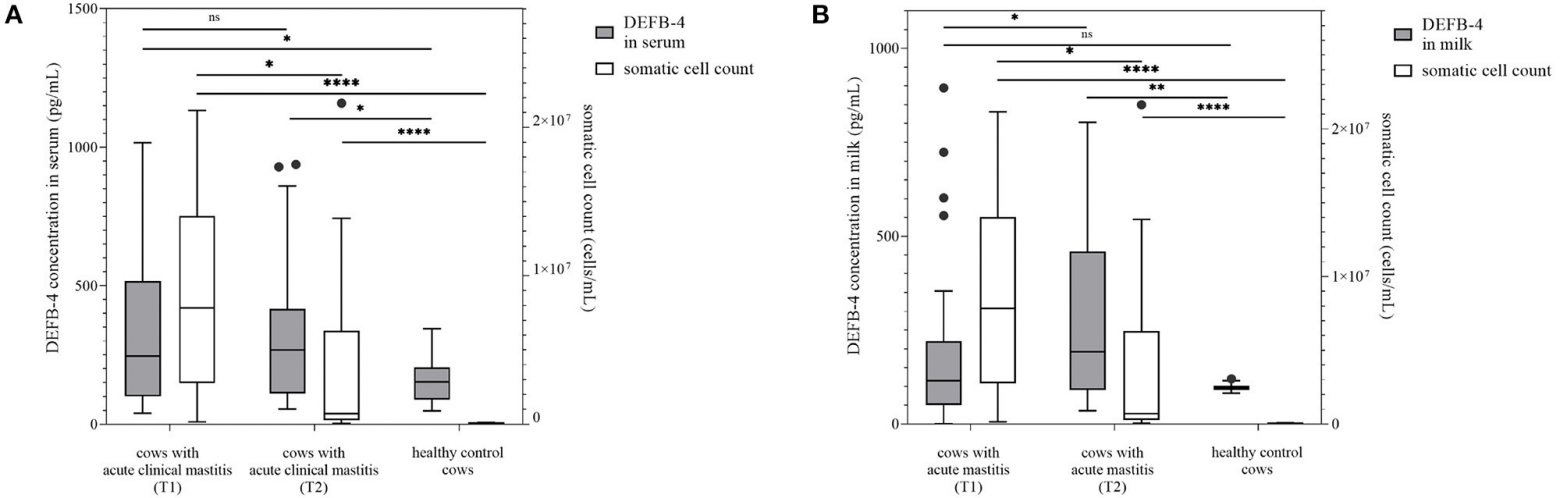
DEFB-4 concentration grouped with the somatic cell count which is currently the standard parameter for the detection of mastitis. 30 cows with acute clinical mastitis at two different times were tested and compared with a healthy control group. T1 marks the time of the initial sampling and T2 the time of the second sampling 12 days later. **(A)** demonstrates DEFB-4 concentrations in serum and **(B)** shows DEFB-4 concentrations in milk. Significant changes are marked by asterisks (*) whereas “ns” means “non-significant”. The significance level is set at *p* < 0.05.

**Figure 2:**
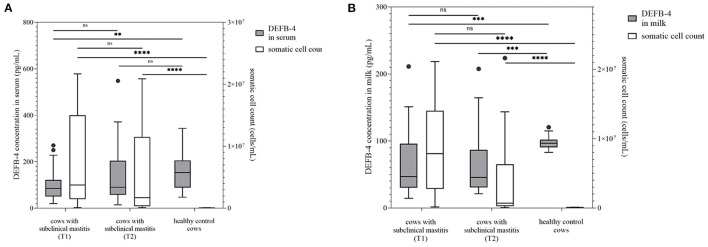
DEFB-4 concentration grouped with the somatic cell count which is currently the standard parameter for the detection of mastitis. 25 cows with subclinical mastitis were tested at two different times and compared with a healthy control group. T1 marks the time of initial sampling and T2 the time of second sampling 12 days later. **(A)** demonstrates DEFB-4 concentrations in serum and **(B)** shows DEFB-4 concentrations in milk. Significant changes are marked by asterisks (*) whereas “ns” means “non-significant”. The significance level is set at *p* < 0.05.

DEFB-4 concentrations in healthy cows were compared with those of diseased cows. Therefore, 30 clinically healthy cows (group 1) whose somatic cell count of milk averaged 44,000 cells/mL (7,000 to 100,000 cells/mL) (Figure 1) were examined and sampled. As expected, animals of this group showed leukocytes within normal range and no signs of disease. In a few samples *Staphylococcus aureus* (*n* = 5), *Streptococcus parauberis* (*n* = 5), *Streptococcus uberis* (*n* = 3) and *Escherichia coli* (*n* = 3) were detected. In this group the DEFB-4 concentrations ranged from 48 to 344 pg/mL (median: 153 pg/mL) in serum and 83–120 pg/mL (median: 97 pg/mL) in milk (Figures 1, 2). Samples from 30 cows with acute clinical mastitis (group 2) were taken. Before sampling it was known due to information of the respective farmer that the combined somatic cell count from all four udder quarters together was increased. However, due to technical problems it was only possible to measure the somatic cell count of the isolated affected udder quarter of 14 cows. Thus, statistical evaluations were based only on 14 animals. The average somatic cell count was 8,890,000 cells/mL (155,000–21,142,000 cells/mL) (Figure 1). All cows of group 2 showed typical clinical symptoms of mastitis. Accordingly, in most samples typical mastitis pathogens were detected, including *S. uberis* (*n* = 12) and *E. coli* (*n* = 6) which were identified as main pathogens. Further details are shown in Supplementary Table 1. In group 2 the highest concentrations of DEFB-4 were found. In serum the concentrations ranged from 40 to 1,016 pg/mL (median: 245 pg/mL), in milk from 0 to 895 pg/mL (median: 115 pg/mL). Compared with DEFB-4 concentrations of the healthy control group, we found that DEFB-4 serum concentrations of group 2 were significantly higher (p = 0.02), but no significance could be detected in milk (*p* = 0.61) (Figure 1).

After 12 days all cows of group 2 were re-examined. A significant decrease of cell count in group 2 was evident (*p* = 0.0105), if only the measurable somatic cell counts were taken into account. The average cell count in milk of 28 measurable samples was 3,836,000 cells/mL (56,000–21,628,000 cells/mL) (Figure 1). *S. uberis* (*n* = 7) and *E. coli* (*n* = 7) could still be detected.

Serum DEFB-4 concentrations ranged from 54 to 938 pg/mL (median: 268 pg/mL) and in milk from 35 to 803 pg/mL (median: 192 pg/mL). A significant difference in DEFB-4 serum concentrations from the initial to the re-examination could not be detected, in milk however, the DEFB-4 concentrations increased significantly (*p* = 0.02) and were now significantly different from the DEFB-4 concentration of healthy cows (*p* = 0.0051) (Figure 1). In summary, the DEFB-4 concentrations in serum differed significantly between healthy cows and cows with acute mastitis at the onset of clinical symptoms, whereas the differences in DEFB-4 concentrations in milk between these groups was only detectable at the later time point (12 days).

### 3.2. Comparison of DEFB-4 concentrations of healthy cows vs. cows with subclinical mastitis

Furthermore, DEFB-4 concentrations of healthy cows (group 1) with 25 cows diagnosed with a subclinical mastitis (group 3) were compared. These cows showed no clinical signs of mastitis, but an increased somatic cell count with an average value of 7,375,000 cells/mL (117,000–21,673,000 cells/mL) (Figure 2), which was not significantly lower than the average value of group 2. Pathogenic bacteria in milk such as *S. uberis* (*n* = 11), *S. aureus* (*n* = 6) and *Streptococcus dysgalactiae* (*S. dysgalactiae*) (*n* = 3) could be found. In group 3 the lowest concentrations of DEFB-4 were detected. In serum the DEFB-4 values ranged from 20 to 271 pg/mL (median: 85 pg/mL), and in milk between 15 and 211 pg/mL (median: 46 pg/mL). DEFB-4 serum and milk concentrations of group 1 were significantly higher than of group 3 (serum: *p* = 0.0025; milk: *p* = 0.0007) (Figure 2). Compared to group 2, in serum and milk the DEFB-4 concentrations of group 3 were significantly lower than those of group 2 (serum: *p* < 0.0001; milk: *p* = 0.015). After 12 days all animals were re-examined. None showed any signs of clinical mastitis. No significant difference between the somatic cell count at the initial and re-examination was detectable (*p* = 0.2522). The average cell count of 24 measurable samples was 5,238,000 cells/mL (101,000–20,880,000 cells/mL) (Figure 2) and *S. uberis* (*n* = 5), *S. aureus* (*n* = 6), *S. dysgalactiae* (*n* = 2) could still be detected. The DEFB-4 values in serum ranged from 15 to 548 pg/mL (median: 90 pg/mL) and 21 and 208 pg/mL (median: 46 pg/mL) in milk. A significant difference in DEFB-4 concentrations from initial to re-examination could not be detected in serum or milk (Figure 2).

Taken together, DEFB-4 levels in healthy cows differed significantly not only from cows with acute mastitis but also from cows with subclinical mastitis, where the difference was detectable in both serum and milk.

**Figure 3:**
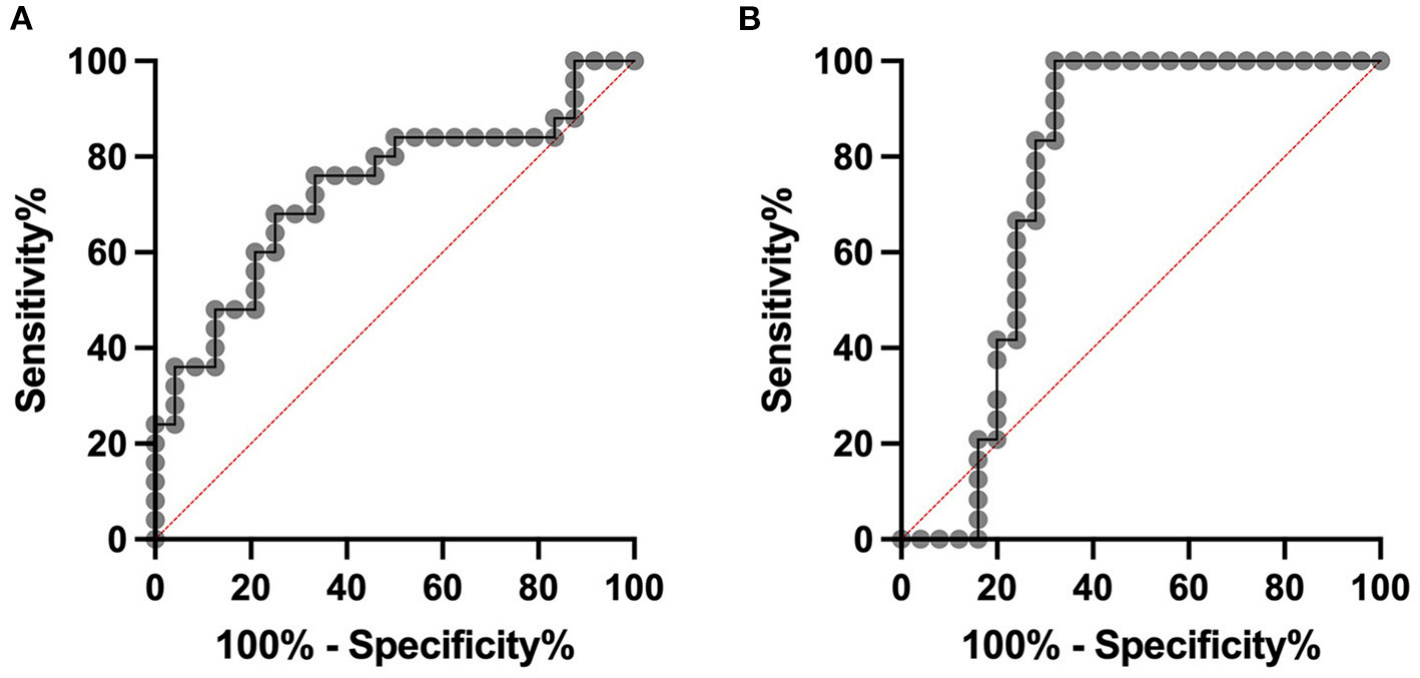
ROC analyses. Cows with subclinical mastitis can be distinguished from healthy controls with a sensitivity of 0.76 and a specificity of 0.62 at a cut-off of 122.4 pg/mL in serum **(A)**. The area under the curve is 0.73. In milk at a cut-off of 89.9 pg/mL sensitivity of 0.83 and a specificity of 0.72 was calculated. The area under the curve is 0.76 **(B)**.

An ROC curve was constructed to distinguish cows with subclinical mastitis and healthy controls. For serum DEFB-4, a sensitivity of 0.76 and a specificity of 0.62 was calculated at a cut-off of 122.4 pg/mL, with an AUC of 0.73 and for milk DEFB-4, a sensitivity of 0.83 and a specificity of 0.72 was calculated at a cut-off of 89.9 pg/mL (Figure 3).

### 3.3. Time kinetics of DEFB-4 in four selected cows over 7 weeks

**Figure 4:**
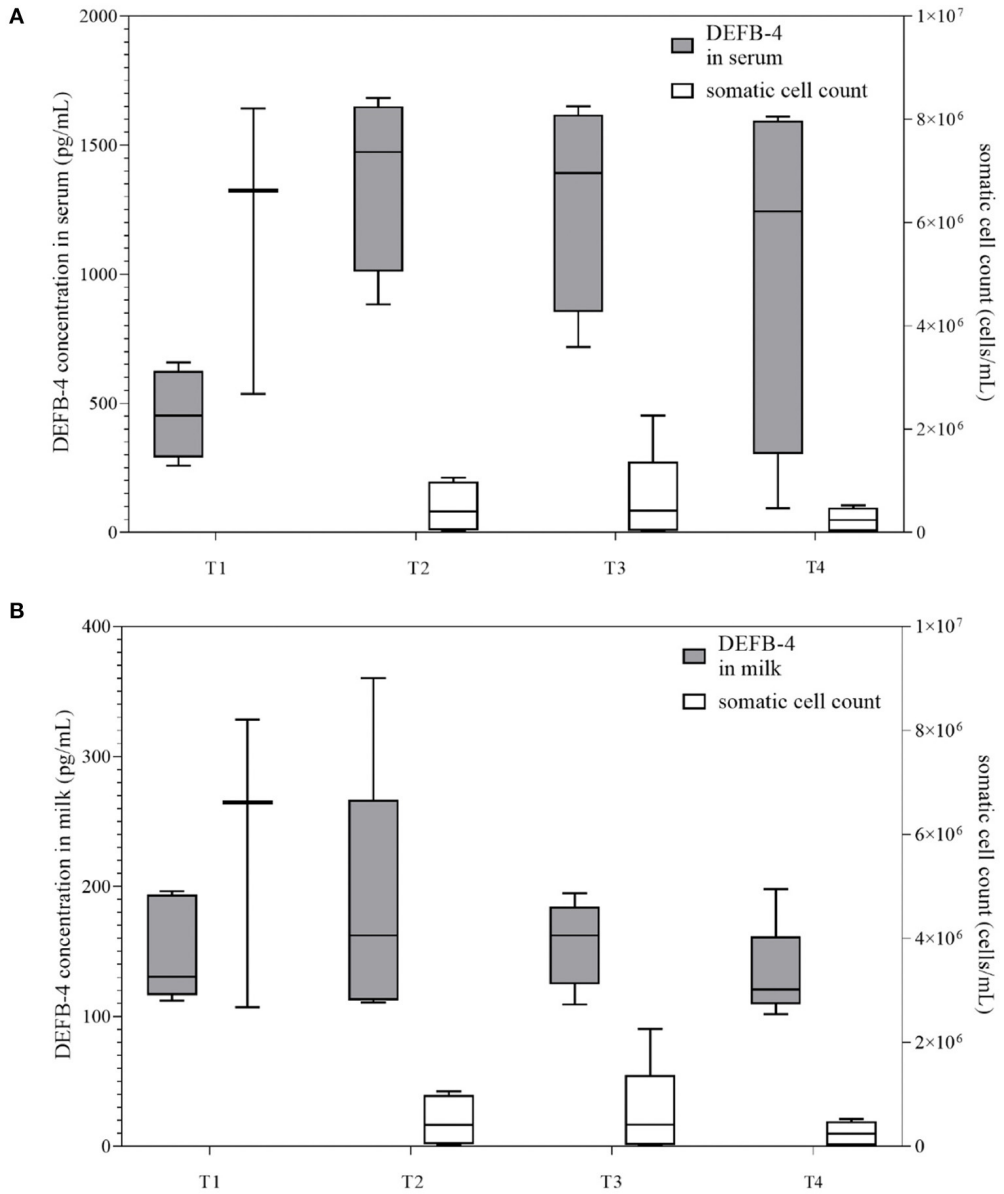
DEFB-4 concentrations of the time kinetics study grouped with the somatic cell count which is currently the standard parameter for the detection of mastitis. Four cows with acute clinical mastitis were sampled directly after the appearance of clinical symptoms and again after 3, 5, and 7 weeks. T1 marks the initial time of sampling. T2, T3, and T4 mark the time of sampling after 3, 5, and 7 weeks. **(A)** demonstrates DEFB-4 concentrations in serum and **(B)** shows DEFB-4 concentrations in milk.

Since DEFB-4 levels in cows with mastitis remained high for at least 12 days, it was interesting to follow DEFB-4 concentrations for a longer time period. Therefore, four cows with acute mastitis were selected, which were sampled directly after the appearance of clinical symptoms and again after 3, 5, and 7 weeks. The average cell count decreased from initial value of 5,832,000 cells/mL to 248,000 cells/mL over 7 weeks, whereas there was no significant change in DEFB-4 concentrations in serum and milk within 3 weeks, when DEFB-4 reached the highest levels. Subsequently the levels decreased, but even after seven weeks they did not reach those of healthy cows (Figure 4). More details are shown in Supplementary Table 2.

### 3.4. *In vitro* study

**Figure 5:**
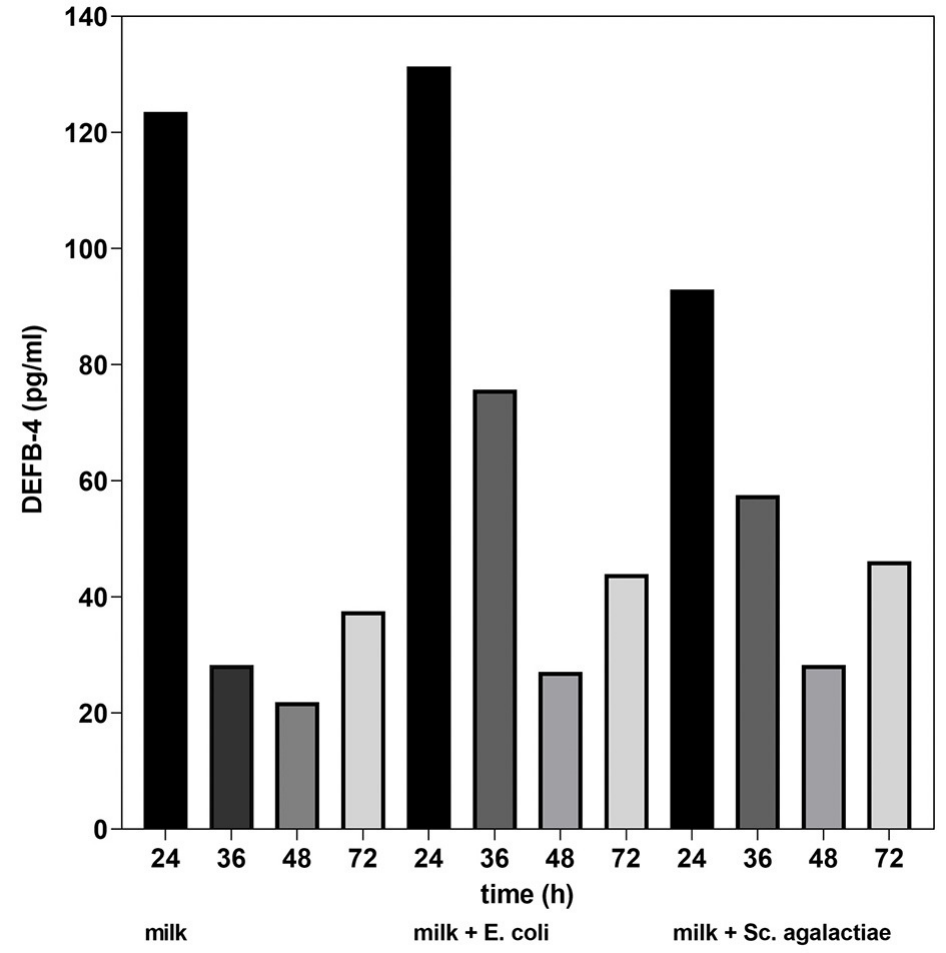
DEFB-4 concentration in milk as part of the *in vitro* study. Comparison of untreated milk, milk + E. coli and milk + Sc. agalactiae.

Together with the first measurement of DEFB-4 the somatic cell count was compared in all four samples. It was 117,000 cells/mL in untreated milk, 115,000 cells/mL in the milk incubated with *E. coli* and 131,000 cells/mL in the milk with *Sc. agalactiae*. The results of the *in vitro* study showed that in untreated cow's milk, there was a significant drop in DEFB-4 concentrations 36 hours after the start of the test series. In the cow's milk incubated with bacteria (E. coli and Sc. agalactiae), the DEFB-4 concentrations also fell after 36 hours, but much more slowly. The lowest value was reached in all groups after 48 h. Thereafter, DEFB-4 concentrations rose again, tending to be somewhat flatter in the untreated milk (Figure 5).

## 4. Discussion

Bacterial mastitis leads to a reaction of the local and systemic immune system. This inflammatory reaction is associated with increased somatic cell count in milk, which is most widely used as a standard diagnostic method. In addition to the primary physical teat barrier, defensins are part of the secondary defense mechanisms to counteract intramammary infections (27). The expression of defensins can be either constitutive or inducible (28). Tetens et al. (29) demonstrated in their study that DEFB-4 is constitutively expressed in the healthy udder, especially in the lymph nodes, although a lower amount was also detected in the glandular epithelium of the cistern and in the udder parenchyma. In humans Tunzi et al. (30) were able to detect β-defensin-1 (hBD-1) in the mammary epithelial cells of healthy, non-lactating women and Jia et al. (31) detected hBD-1 in human breast milk. In case of an infection, Goldammer et al. (7) found that bovine DEFB-5 mRNA is expressed in mammary epithelial cells of the affected glandular tissue in cows with mastitis. The results of our study also show that DEFB-4 levels can be detected in healthy cows in milk and serum, indicating constitutive expression at low levels. In cows with acute mastitis much higher DEFB-4 levels were detected, which is an indication for induced expression. In most of the studies defensins are detected on a genetic level and it could be proved that defensin genes are strongly upregulated in case of mastitis. Only one study (32) detected lingual antimicrobial peptide (LAP) by ELISA, which is also a defensin in cows. A method for detection of DEFB-4 at a protein level could contribute to the development of a rapid test. As this is very important for everyday practice, the decision was made to use this measurement method for our study. The results of our study show that bovine DEFB-4 protein levels can be measured locally in milk as well as systemically in serum with ELISA and that there is a correlation between the DEFB-4 concentration and the severity of mastitis. DEFB-4 levels in milk and serum of healthy control cows, of cows with acute clinical mastitis and of cows with subclinical mastitis were compared. The DEFB-4 values of an initial examination were also compared with those of a re-examination 12 days later.

Comparing the DEFB-4 concentrations within the three groups, it is apparent that in cows with acute clinical mastitis the DEFB-4 concentration was significantly highest in serum. In milk however, the local defensin concentration of cows with acute mastitis was not significantly higher than those of the healthy control group. Our hypothesis is that this could be due to delayed local defensin expression. Cows with acute mastitis were sampled immediately after the onset of clinical symptoms, which could presumably be a time before the increase in local defensin production. However, increased defensin concentrations were already detectable in serum at this time. It concludes that the response to the mastitis pathogens initially leads to a systemic defensin release and subsequently to a local increase in defensin production. It is not clear whether the local increase is also caused by systemic defensin or whether the systemic defensin should prevent the bacteria from migrating. Cows with subclinical mastitis showed lower concentrations of defensin in serum and milk than healthy control cows and those with acute mastitis. Therefore, we suppose that subclinical mastitis initially leads to a consumption of constitutively expressed defensins before an induction of defensin expression occurs. As far as we know this could not be confirmed by other studies.

The results of our *in vitro* study show that the DEFB-4 concentration in milk decreases over time, which we believe is due to cell death. For example, the lifetime of neutrophil granulocytes in tissue is about 1–2 days before they undergo a spontaneous apoptosis (33). The addition of mastitis pathogens significantly reduced the decrease in DEFB-4 levels, which we believe confirms the induction of defensin expression by pathogens. After 48 hours, the defensin concentration increased again in each sample. Since most neutrophil granulocytes must have died by this time, we conclude that not only neutrophil granulocytes but also other somatic cells express defensin.

The results of the re-examinations after 12 days show that the animals of both mastitis groups are not fully recovered. 10% of the animals of the acutely ill animals still show a poor general condition. The somatic cell count decreased but is still significantly above the reference of 100,000 cells/mL. DEFB-4 milk concentrations in cows with acute mastitis increase further at the time of re-examination after 12 days, which leads us to conclude that local defense was still active. Although the time of re-examination was consistent with the course of acute mastitis reported in the literature, according to which acute mastitis is treated for ~3–8 days (34, 35), the results of our study show that the immune system reacted to the infection for much longer. Even the results of DEFB-4 levels over a period of 7 weeks of four animals with an acute clinical mastitis did not indicate complete recovery. DEFB-4 levels were still higher than those of healthy control cows which could mean that the immune system is still reacting to the infection. Our results reflect that the active disease process in acute and subclinical mastitis lasts longer than previously thought. Fogsgaard et al. (36) also conclude that the cows suffering from an acute clinical mastitis have not fully recovered even 8 weeks after antibiotic treatment. Since the somatic cell count, which is currently the standard parameter for the detection and monitoring of the course of mastitis, has decreased much faster than DEFB-4 concentrations, it is assumed that DEFB-4 is much more appropriate to make a prediction about the immune status of the animals.

In addition to the correlations of DEFB-4 levels studied within the groups and at different points in time, also it is investigated whether there are correlations of DEFB-4 levels with the somatic cell count, the internal body temperature and the leukocytes. In none of the investigations a significant correlation could be found. The classification into the three groups of this study cannot be reconstructed and confirmed by the somatic cell count, the internal body temperature or the leukocytes, because the results of these parameters do not correlate with the results of the clinical examination. Usually the somatic cell count is standardly used to detect mastitis in cows, but sometimes it is technically not measurable due to altered pH values or precipitated proteins and therefore it is not always reliable. In addition, the somatic cell count cannot be used to distinguish acute mastitis from subclinical mastitis. Furthermore, the pathogens that caused mastitis are examined and compared to the DEFB-4 levels. In this study, mainly *S. uberis* and *E. coli* are detected as mastitis causing pathogens in cows with acute mastitis. This distribution corresponds to the results of other scientific studies (22). Mastitis pathogens are also detected sporadically in the group of healthy animals. They are considered as contaminants since the animals do not develop mastitis and the somatic cell count remained <100,000 cells/mL. Petzl et al. (37) found that the pathogen species affect the course of mastitis and that *E. coli* is responsible for an early increase in β-defensins. Günther et al. (38) and Younis et al. (21) describe similar findings. In the present study only a tendency of elevated DEFB-4 concentrations in *E. coli* infections could be demonstrated, but it has not been seen any significant correlations with the type of pathogens. This topic would have to be addressed again in a future study with statistically relevant sample numbers. One reason for the increased amount of DEFB-4 in *E. coli* infections could be that due to the molecular structure of *E. coli* the depolarization of the membrane and thus the lysis of the pathogen is more difficult for DEFB-4 and therefore the body reacts with a higher amount of defense parameters. The problem does not lie in the detection of the *E. coli* pathogen, since the increase of DEFB-4 is extremely fast.

Most mastitis patients today are treated with antibiotics (39). Based on the clinical examination, which is highly subjective, all acutely ill animals from our study are treated with antibiotics, while the subclinically ill animals were not treated. This decision can be supported by the more objective parameter DEFB-4, as its concentrations allow to comprehend the classification of the animals into the three groups of our study. Based on the ROC analysis we are able to calculate cut-off values for differentiation of the groups. A sensitivity of 0.83 in milk shows that a decision about the treatment of mastitis is possible based on the DEFB-4 concentration. If future studies of DEFB-4 levels with higher sample numbers confirm our results, it could be possible that DEFB-4 concentrations can be used to estimate the necessary treatment strategy. However, since the groups sometimes overlap considerably, it must be remembered that a clinical examination can never be replaced.

Since the resistance of pathogens to antibiotics is steadily worsening (40), it is even more important to rely on endogenous immune defense mechanisms. Tunzi et al. (30) hypothesized that women who express less hBD-1 hereditarily have a higher risk of bacterial colonization. One approach in the future could be to influence the amount of DEFB-4 by husbandry and breeding or the *in vitro* production of DEFB-4 in order to use it as a natural antibiotic.

As limitations of this study, single animal effects have to be considered. The study of time kinetics would also need to be continued with higher sample numbers of cows for completion to determine the time of full recovery.

## 5. Conclusion

Defensins are known as multifunctional peptides, which have both an antibacterial and immunomodulatory effect and therefore it can be assumed that they play an important role in the immune defense of mastitis. The present study supports this statement as a correlation between the severity and course of mastitis and the measured DEFB-4 concentration could be shown. With regard to DEFB-4 levels, the results of this study show a significant difference between acute clinical mastitis and subclinical mastitis, both locally and systemically. Clinically manifest mastitis could therefore be identified with DEFB-4 and treated if future studies can confirm our results. Since subclinically affected animals could be significantly differentiated from the healthy control group in serum and milk, DEFB-4 could also be used as a marker for the detection of subclinically affected animals. It could be possible to detect DEFB-4 swiftly in the future using a rapid test to determine acute and subclinical mastitis in cows and maybe it is possible to reduce the amount of antibiotics in the future since many cows with subclinical mastitis are treated unnecessarily nowadays with antibiotics just because the somatic cell count is too high.

## Data availability statement

The original contributions presented in the study are included in the article/Supplementary material, further inquiries can be directed to the corresponding author.

## Ethics statement

The animal study was reviewed and approved by Lower Saxony State Office for Consumer Protection and Food Safety (Niedersächsisches Landesamt für Verbraucherschutz und Lebensmittelsicherheit, LAVES) (File number: 33.9-42502-05-16A089).

## Author contributions

Conceptualization, formal analysis, writing—original draft preparation, supervision, and project administration: SN. Methodology: SN and AF. Investigation: AF. Writing—review and editing: SS. Visualization: SN and SS. All authors have read and agreed to the published version of the manuscript.
