# Supplementary material for: β-defensin-4 as an endogenous biomarker in cows with mastitis
Source: Front Vet Sci. 2023 Mar 24;10:1154386. doi: 10.3389/fvets.2023.1154386 (PMC10079942; doi:10.3389/fvets.2023.1154386)
Supplement: Supplementary file 1 [file Data_Sheet_1.docx]

Supplementary Material

β-DEFENSIN-4 AS AN ENDOGENOUS BIOMARKER IN COWS WITH MASTITIS

S. Neumann*, S. Siegert, A. Fischer

*** Correspondence:** Stephan Neumann: sneuman@gwdg.de

Supplemental Table 1. Summary of gathered details of each group

|  | Cows with clinical mastitis | | Cows with subclinical mastitis | | Healthy control cows  T1 |
| --- | --- | --- | --- | --- | --- |
|  | T1 | T2 | T1 | T2 |  |
| internal body temperature (°C) | 39.2 (38.3 to 40.8) | 38.6 (38.1 to 41.1) | 38.5 (37.6 to 38.9) | 38.6 (37.8 to 38.9) | 38.3 (37.3 to 38.9) |
| somatic cells / mL (n = measurable samples) | 7,842,000  (n=14) (155,000 to 21,142,000) | 701,000 (n=28) (56,000 to 21,628,000) | 3,919,000 (n=25) (117,000 to 21,673,000) | 1,700,000 (n=24) (101,000 to 20,880,000) | 36,000 (n=30) (7,000 to 100,000) |
| leukocytes (10^3^ / µl) (n = measurable samples) | 6.7 (n=24) (0.4 to 13) | 9.4 (n=30) (0.2 to 27.5) | 8.6 (n=24) (5.4 to 17.9) | 8.0 (n=24) (3.5 to 11.4) | 7.0 (n=29) (4.7 to 10.0) |
| bacteria (main pathogens) | *Streptococcus uberis* (n=12), *Escherichia coli* (n=6) | *Streptococcus uberis* (n=7), *Escherichia coli* (n=7) | *Streptococcus uberis* (n=11), *Staphylococcus aureus* (n=6), *Streptococcus dysgalactiae* (n=3) | *Streptococcus uberis* (n=5) *Staphylococcus aureus* (n=6), *Streptococcus dysgalactiae* (n=2) | *-* |

T1 marks the initial time of sampling, T2 the time of sampling after 12 days.

Supplemental Table 2*.* Summary of gathered details of 4 selected cows with clinical mastitis over 7 weeks

|  | clinical mastitis | | | |
| --- | --- | --- | --- | --- |
|  | T1 | T2 | T3 | T4 |
| internal body temperature (°C) | 38.6 (38.1 to 39.5) | 38.2 (37.9 to 38.4) | 38.2 (38.1 to 38.5) | 38.1 (37.8 to 38.3) |
| somatic cells / mL (n = measurable samples) | 5,832,000 (n=3) (2,676,000 to 8,208,000) | 492,000 (n=5) (20,000 to 1,059,000) | 645,000 (n=5) (23,000 to 2,261,000) | 248,000 (n=5) (16,000 to 524,000) |
| leukocytes (10^3^ / µl) | 6.6 (3.1 to 8.3) | 9.0 (5.8 to 12.6) | 7.8 (6.5 to 9.4) | 7.8 (6.1 to 8.9) |
| bacteria (main pathogens) | *Staphylococcus aureus* (n=3), *Streptococcus uberis* (n=2), *Streptococcus dysgalactiae* (n=1), *Escherichia coli* (n=1),  *Klebsiella oxytoca* (n=1) | - | *Streptococcus parauberis (n=1)* | *Staphylococcus aureus* (n=2), *Streptococcus uberis* (n=1) |

T1 marks the initial time of sampling. T2, T3 and T4 mark the time of sampling after 3, 5 and 7 weeks.
